# Preparation of Glass-Ceramics Using Zinc-Containing Smelting Slag: Structure, Properties and Solidification of Zinc

**DOI:** 10.3390/ma18153555

**Published:** 2025-07-29

**Authors:** Nannan Wu, Junhui Huang, Junxi Qiu, Zonghang Li, Xiaofan Li, Bohan Li, Nianzhe Li, Yuxuan Zhang, Shunli Ouyang

**Affiliations:** 1 School of Arts and Sciences, Guangzhou Maritime University, Guangzhou 510725, China; woshinannan04@163.com (N.W.); ldu2013yuxuan@outlook.com (Y.Z.); 2 School of Shipping, Guangzhou Maritime University, Guangzhou 510725, China; hjh20050222@163.com (J.H.); 17512900436@163.com (J.Q.); 17686215263@163.com (Z.L.); xiaofanelvali@163.com (X.L.); 13054226527@163.com (B.L.); 3 School of Material and Metallurgy, Inner Mongolia University of Science & Technology, Baotou 014010, China; 13598707766@163.com

**Keywords:** Raman spectroscopy, slag glass-ceramics, heavy metals, solidification, solid waste

## Abstract

The stabilization of heavy metal elements, such as zinc, in the form of ions within the glass-ceramics represents a valuable approach to addressing environmental pollution caused by heavy metals. This study investigates the feasibility and physicochemical properties of diopside-based glass-ceramics synthesized from zinc-containing smelting slag. The zinc-rich smelting slag is abundant in SiO_2_, Al_2_O_3_, CaO, and other constituents, thereby providing cost-effective and efficient raw materials for glass-ceramic production. The conversion of zinc-containing smelting slag into glass-ceramics was achieved through a melting process. We analyzed the effects of varying doping levels on the properties of the resulting glass-ceramics. The results indicated that as the doping level of smelting slag increases, the crystallization temperature of the glass-ceramics decreases while the crystal phases of diopside and anorthite progressively increase, significantly enhancing both mechanical strength and chemical stability. Notably, when the doping level reaches 60%, these glass-ceramics exhibit remarkable physical properties, including high density (3.12 g/cm^3^), Vickers hardness (16.60 GPa), and excellent flexural strength (150.75 MPa). Furthermore, with increasing amounts of doped smelting slag, there are substantial improvements in acid resistance, alkali resistance, and corrosion resistance in these materials. Raman spectroscopy and EDS analysis further verified a uniform distribution of the crystal phase and effective immobilization of heavy metal zinc.

## 1. Introduction

In recent years, industrial activities have increased continuously, and the treatment and reuse of industrial byproducts and wastes have become the focus in the field of environmental science and material engineering. Zinc production market ((a) 2024 USD 29.73 billion; 2032 USD 64.10 billion; https://www.analytica.global/research/zinc-mining-market, 12 June 2024), (b) zinc recycling market zinc recycling market size was valued at USD 10.9 billion in 2023 and is projected to reach USD 15.7 billion by 2030, growing at a CAGR of 6.9% during the forecasted period 2024 to 2030. Zinc-containing smelting slag, a typical byproduct of the metallurgical industry, presents a complex composition and contains harmful substances such as heavy metals. Traditional disposal methods, such as landfilling and stockpiling, not only consume substantial land resources but also pose a risk of severe environmental contamination [1]. Therefore, the development of new utilization strategies to transform these wastes into high-value materials is a hot topic of current research. Slag glass-ceramics represent a novel class of environmentally friendly materials. Through controlled cooling and heat treatment processes, highly uniform microcrystalline phases can be formed within the glass matrix, thereby significantly improving its physical and chemical properties [2,3]. Compared to conventional glass, glass-ceramics exhibit superior mechanical strength, wear resistance, and chemical stability [4,5]. Diopside is the main crystalline phase in glass-ceramics, has been widely studied due to its excellent physicochemical properties [6,7].

On the reuse of zinc-containing smelting slag, studies have shown that the rich SiO_2_, Al_2_O_3_, CaO, and other components in these minerals make them ideal raw materials for the preparation of glass-ceramics. Pi et al. [8] found that with the increase in heavy metal zinc content, zinc was stably solidified in glass-ceramics in the form of zinc silicate phase, and its leaching concentration was much lower than the standard limit. Li et al. [9] adjusted the proportion of heavy metals zinc and zirconium that changed the crystal phase of glass-ceramics (ZrO_2_ and (MgAl_2_Si_3_O_10_)_0.6_ phase disappeared, Zn_2_Al_4_Si_5_O_18_ and ZnAl_2_O_4_ phase appeared) and promoted the crystallinity, significantly improving its mechanical properties and dielectric properties. Furthermore, Pei et al. [10] propose an innovative boiling furnace roasting process for the efficient recovery of zinc (93.38% removal) and lead (98.14% removal) from electric arc furnace dust (EAFD) and coke dry quenching dust (CDQD), achieving high-grade crude zinc (87.58% Zn) and iron-rich alloy slag (52.38% Fe) while addressing environmental challenges through low-temperature (1050 °C) and energy-efficient fluidized-bed reduction, validated by thermodynamic, kinetic, and experimental analyses.

The presence of zinc not only facilitates the formation of crystal nuclei but also contributes to the cure of heavy metals and reduces environmental pollution through the formation of stable crystalline phases [11,12]. Furthermore, the energy consumption associated with the fabrication of the glass-ceramics is relatively low and contributes to economic and environmental objectives [13]. However, the high chemical activity and complexity of zinc-containing slag pose challenges. How to effectively control the crystallization process of glass-ceramics, optimization of performance, and ensuring the stable solidification of heavy metals has become a key issue in current research [14,15]. This study not only highlights the potential application of zinc-containing smelting slag in producing glass-ceramics but also offers a novel technical pathway alongside theoretical support for resource utilization strategies concerning smelting slags. By studying the microstructure and performance relationship of glass-ceramics in detail, we can better understand the characteristics of these materials and provide a theoretical basis for their industrial applications [16].

## 2. Experimental Section

### 2.1. Sample Preparation Process

In the study, the main crystal phase was determined based on the compositional characteristics of zinc-containing smelting slag and in conjunction with the ternary phase diagram of the CaO-MgO-SiO_2_ system. Glass-ceramics were fabricated using the melting method. The physical and chemical properties of glass-ceramics were mainly determined by the internal chemical composition and microstructure. In contrast to amorphous conventional glass, the atomic arrangement within glass-ceramics showed a certain degree of regularity, and the components had distinctive properties. The feature necessitates control of the component proportions within a specified range during the preparation of glass-ceramics.

**Figure 1:**
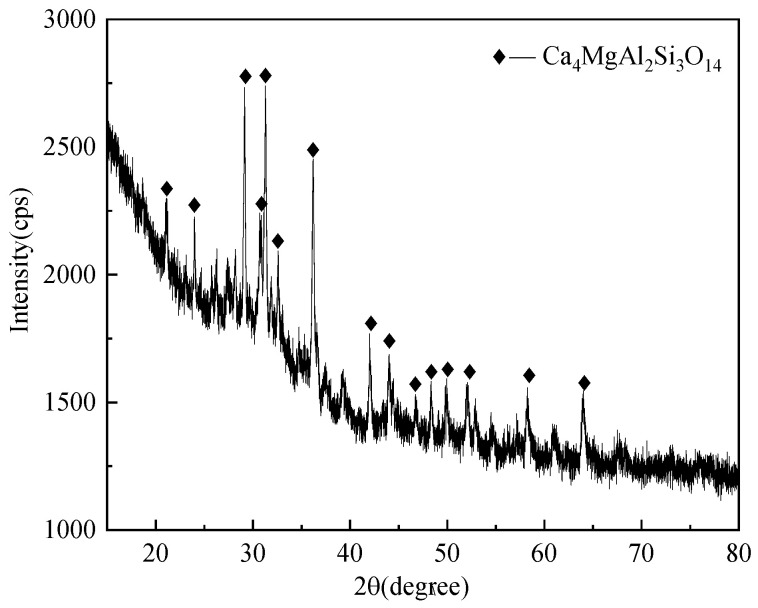
XRD pattern of zinc-containing smelting slag.

Table 1 shows the chemical composition of zinc-containing smelting slag. The chemical composition is diverse, providing a rich selection of raw materials for the manufacturing of glass-ceramics. The smelting slag contains various elements such as silicon, aluminum, iron, and calcium, which are the main components required for manufacturing glass-ceramics. Therefore, using smelting slag to manufacture glass-ceramics not only has an abundant provision of raw materials but also could adjust formulas based on the chemical composition of the slag. It produces glass-ceramics with different properties and applications. Furthermore, the physical properties of smelting slag make it an ideal raw material for manufacturing glass-ceramics. These metallurgical slags have fine particle size and good uniformity, which is extremely beneficial for the formation and crystallization process of glass-ceramics. Using the raw materials, one could control the structure and performance of glass-ceramics easily, achieving higher product quality and more extensive application fields. The XRD pattern of the smelting slag is depicted in Figure 1.

**Figure 2:**
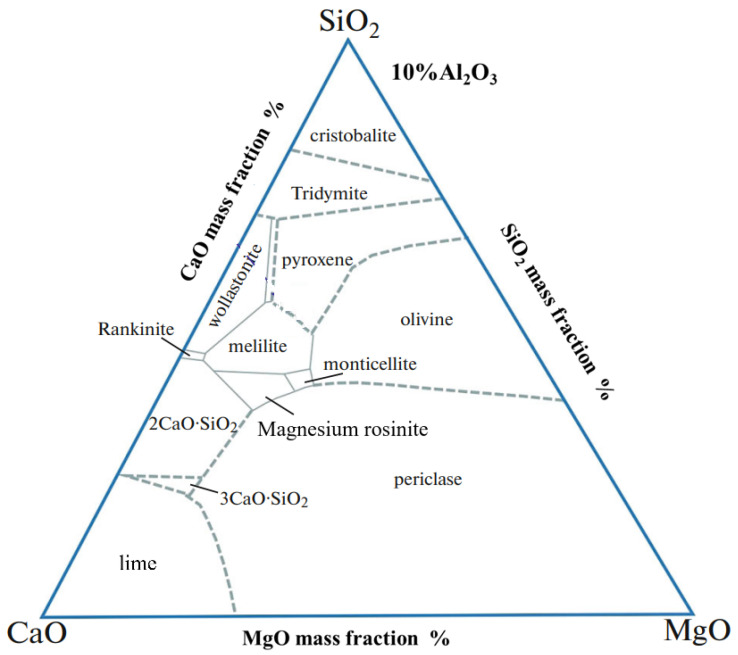
Phase diagram of CaO-Al_2_O_3_-SiO_2_(Al_2_O_3_-10%) system glass [18].

The study indicated that zinc-containing smelting slag was utilized to prepare glass-ceramics, and glass-ceramics obtained diopside as the main crystal phase [17]. The CaO-MgO-Al_2_O_3_-SiO_2_ (CMAS) system glass-ceramics with diopside as the main crystal phase have good acid and alkali resistance, flexural strength, hardness, abrasion and erosion resistance, etc. The formula was determined by the ternary phase diagram of CaO-Al_2_O_3_-SiO_2_ containing 10 wt.% alumina (Figure 2). Glass-ceramics with better performance can be produced to optimize formula by adding quartz sand and sodium carbonate.

Slag glass-ceramics were prepared by designing the chemical composition of basic glass with different doping amounts of smelting slag, and their composition is shown in Table 2. Table 2 shows that the slag produced by China Metallurgical Group Corporation is from Baotou City, Inner Mongolia.

**Figure 3:**
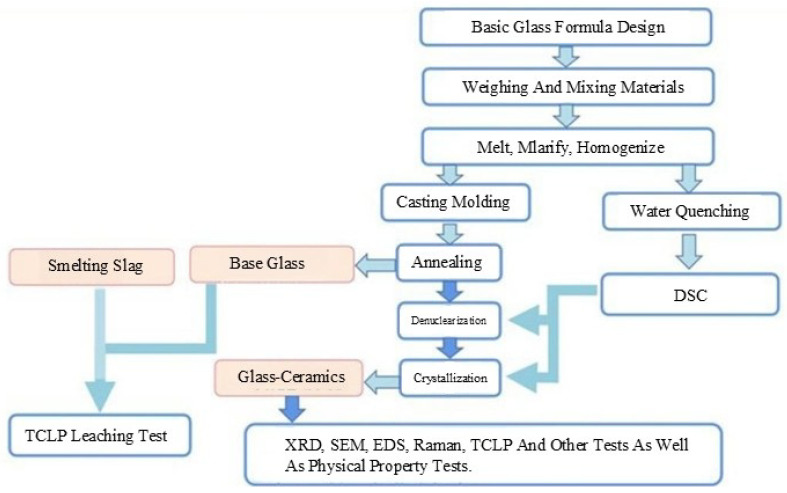
Experimental process.

In the experiment, reagents such as quartz sand (>99%), CaO (>99%), MgO (>99%), and Al_2_O_3_ (>99%) were used for supplementation to make up for the deficiency of the main components of CMAS glass. The influence of zinc, the most common heavy metal in the smelting slag, on the structure and properties of glass-ceramics was studied using zinc-containing smelting slag as the main raw material. The flowchart of the experiment is depicted in Figure 3.

(1) Ingredients: Weigh the corresponding raw materials based on the formula.
(2) Mixing: Put the weighed ingredients into the jar mill and run it at a speed of 150 revolutions per minute for 30 min to ensure thorough mixing of the ingredients.
(3) Molten water quenching: Place the well-mixed ingredients in a corundum crucible, place in a high-temperature resistance furnace, and melt at 1550 °C for 3 h, and quickly pour the molten glass liquid into cold water for water quenching to obtain molten water quenching slag. After the molten water quenching slag is crushed and passed through a 200-mesh sieve, DSC testing can be conducted. Correspondingly, the subsequent heat treatment system of the sample can be further determined.
(4) Casting molding: The uniform melt is rapidly poured onto a metal mold to obtain glass, which is transferred to a preheated annealing furnace at 600 °C for annealing for 10 h to reduce residual internal stress. Subsequently, it is cooled to room temperature in the furnace and taken out.
(5) After cutting the base glass into regular shapes of specific sizes, the glass samples can undergo further heat treatment and testing.

### 2.2. Testing Method

The water-quenched sample of the base glass obtained after annealing was ground into fine powder (particle size < 200 μm), and 1 g was examined by differential scanning calorimetry. The differential scanning calorimeter (DSC, STA-449C, Netzsch-Gerätebau GmbH, Selb, Germany) was heated at 10 °C/min from room temperature to 1200 °C in nitrogen. The glass transition temperature T_g_ and the exothermic peak temperature Tc of crystallization of glass-ceramics with different chemical compositions were determined to determine the heat treatment process. The reference substance was high-purity alumina powder; the temperature error is ±5 °C.

The leftover scraps of the glass-ceramics cutting sample strips were ground into powder with an agate mortar. After grinding, they were passed through a 200-mesh (74 μm) sieve. The phase composition of the samples was determined by X-ray diffraction (XRD, X ‘Pert Pro Powder, Xpert Professional, Belfast, UK). The radiation source used Cu as the target material with a scanning rate of 10°/min, in the scanning range from 10 to 80 degrees.

The glass-ceramics samples were cut into small rectangular prisms with specifications of 3 × 4 × 6 (mm). Find a smooth surface with no visible defects to the naked eye, and coarse-grind step by step with metallographic sandpaper of 600 to 2000 grit. Then, a 0.25 μm diamond spray polishing agent was selected on the polishing machine for polishing until there were no scratches on the surface, and a mirror surface was presented. The microstructure of the glass-ceramics was characterized by a scanning electron microscope (SEM, Supra 55 FESEN, Zeiss, Oberkochen, Germany).

The prepared glass-ceramics samples were etched with a 5% molar mass hydrofluoric acid solution for 60 s and then cleaned with ultrasonic waves and ethanol. Gold was sprayed on the surface of the sample to obtain good electrical conductivity. The glass-ceramics samples were cut into small rectangular prisms, and Raman analysis was conducted on a flat surface without obvious defects such as pores. The Raman spectrometer used in the experiments was a Renishaw in Via-Qontor Raman spectrometer (Renishaw, Wotton-under-Edge, UK), with excitation wavelength of 532 nm, a 1200 lines/mm grating, an exposure time of 10 s, a cumulative number of 7 times, and Raman spectral data acquisition range (100–1500 cm^−1^).

The dielectric properties were measured using a precision impedance analyzer (Agilent 4294A, Agilent Technologies, Santa Clara, CA, USA) at ambient temperature to measure, with a frequency range of 40 Hz to 100 MHz. Both surfaces of the sample were polished and coated with silver to enhance the contact with the electrode. The dielectric constant ε and the dielectric loss factor tanδ were obtained through measurement.

The Vickers hardness was determined by using the microhardness tester (DHV-1000, Beijing Shangliuguang Instrument Co., Ltd., Beijing, China). At least 5 points were measured for each sample to obtain the average hardness, with an error of ±0.2 GPa. The flexural strength of the sample 3 × 4 × 40 (mm) was tested by the three-point bending method using the CSS-44100 universal testing machine (Jinan Ruima Mechanical Equipment Co., Ltd., Jinan, China), and the measurement speed was 0.5 mm/min. The volumetric density was measured by Archimedes’ method.

Based on the “Test Methods for Performance of Cast Stone Products-Acid and Alkali Resistance Tests” of JC/T 258-1993 [19], glass-ceramics particles with a particle size of 0.5 to 1.0 mm were heated in a 100 °C water bath and subjected to a corrosion test for 1 h and using 20% NaOH and 20% H_2_SO_4_, respectively, to determine the acid and alkali resistance of the samples.

According to the standard “HJ/T 299–2007” [20], the leaching concentration of heavy metals was determined. The size of specimens was less than 200 grit (74 μm), the pH = 3.2, and the liquid–solid ratio was 10:1 (L/kg). Put the sample into the shaking flask and use a mixture of concentrated sulfuric acid and concentrated nitric acid as the buffer solution.

## 3. Results and Discussion

### 3.1. Differential Scanning Calorimetry

**Figure 4:**
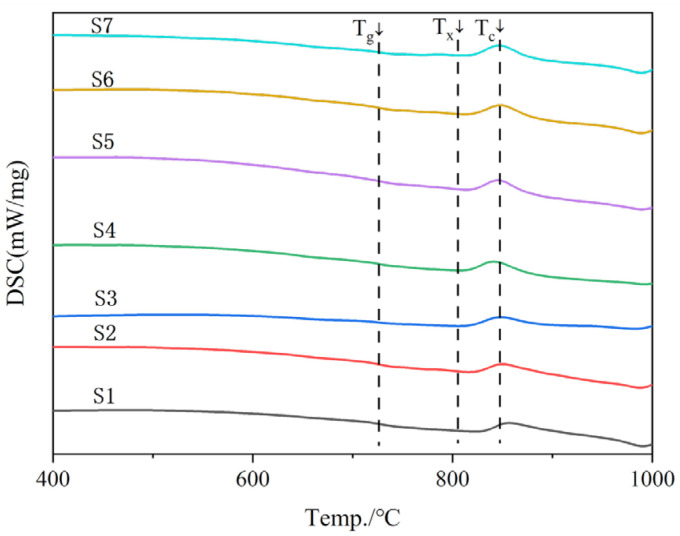
DSC curves of the glass with different contents of smelting slag.

Figure 4 shows the DSC curves of water-quenched samples of seven glass matrices after melting. The endothermic peaks were not obvious in seven samples from S1 to S7. All the curves had an exothermic peak. It was the phase transition or endothermic and exothermic reactions produced from the samples in the temperature changed. The glassy substances have higher internal energy than their corresponding crystalline substances from the perspective of thermodynamics. They can release energy through crystallization or phase separation under certain conditions. The endothermic peak is not obvious due to the glass crystallizing below the glass transition temperature (T_g_). Figure 4 shows that with the increase in the content of zinc-containing smelting slag, the peak temperature of exothermic for the crystallization of glass decreased from 868 °C to 841 °C. The experimental phenomenon is due to the increase in the content of Zn^2+^, which then forms more nucleation sites, resulting in gradual temperature decreases in the exothermic peak during crystallization, and the crystals are easier to precipitate [21]. There are small amounts of nucleating agents in the raw materials, such as zinc oxide, titanium dioxide, chromic oxide, rare and precious metals, etc. Certain temperatures allow the prepared glass-ceramics to undergo crystallization. The crystallization of the sample will make an exothermic phenomenon, and the exothermic peak is not obvious, indicating that the crystallization degree of the sample is not high [22]. The heat treatment conditions of the samples were determined based on the DSC results, as shown in Table 3. The nucleation temperature of glass-ceramics is taken as 50 °C above T_g_, the crystallization temperature is taken as the peak temperature of heat release during crystallization (T_c_), and both the nucleation and crystallization times are 4 h.

### 3.2. Phase Composition Analysis of Slag Glass-Ceramics

**Figure 5:**
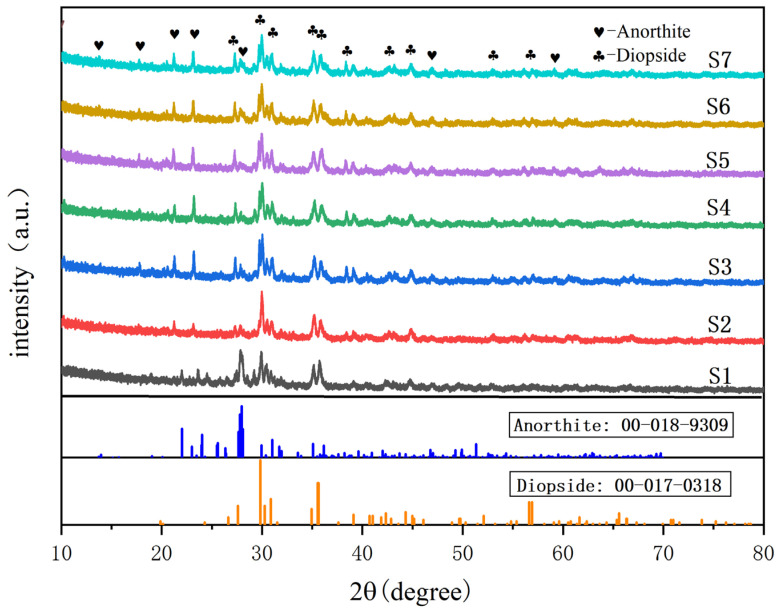
XRD patterns of glass-ceramics with different content of smelting slag.

Figure 5 shows the XRD patterns of glass-ceramics with different contents of smelting slag after heat treatment at different nucleation and crystallization temperatures. The experimental results indicate that the main crystal phases of the seven groups of samples are all diopside phases (CaMgSi_2_O_6_), and the intensities and positions of the diffraction peaks of samples with different components are almost the same. The increase in the doping amount of the smelting slag causes the crystal phase type of the sample to change, accompanied by the second phase calcium feldspar phase (CaAl_2_Si_2_O_8_), and the intensity of the diffraction peak keeps increasing. Zinc-containing smelting slag contains nucleating agents that promote crystallization, such as ZnO, TiO_2_, etc. [23]. These nucleating agents play a nucleating role and increase the fluidity at the interface [24], and promote crystal growth. With an increase in the doping amount of the smelting slag let the content of the nucleating agent increases accordingly, reducing the crystallization activation energy of the sample and thereby promoting its crystallization. The diffraction peaks of the main crystal phase of the sample are mutually confirmed with the exothermic peaks of crystallization in the above DSC results, showing that as the doping amount of the smelting slag increases, T_c_ and ΔT decrease, and the crystal phase is more likely to precipitate. Based on the XRD data from samples S1 and S2, the reduction in silicon dioxide (SiO_2_) content from 6% to 2% induces a shift in the diffraction region from 25° to 32°, indicating a significant alteration in the proportion of the main crystalline phase.

### 3.3. Microstructure Analysis of Glass-Ceramics

**Figure 6:**
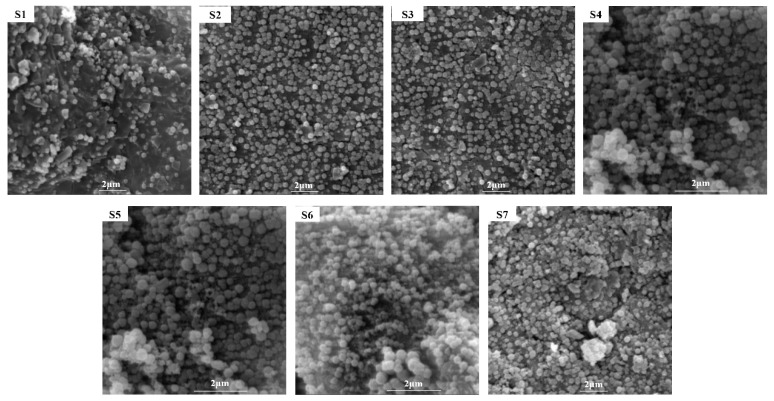
SEM images of slag glass-ceramics with different smelting slag content.

Figure 6 shows the SEM images of slag glass-ceramics with different content of smelting slag. The addition of zinc-containing smelting slag has a certain influence on the crystal distribution and morphology of slag glass-ceramics. As shown in Figure 6, dense spherical crystals are uniformly distributed in the glass matrix of the slag glass-ceramics. The content of smelting slag is relatively small, making the slag glass-ceramics composed of the initial phase, diopside phase, and the residual glass phase, and the main crystal phase being the diopside phase. As the increase in the content of smelting slag let the density of diopside phase spherulites increases significantly, while the proportion of the internal glass phase decreases. The doping amount of the smelting slag exceeds 55 wt.% makes the number of grains increase significantly, the contours are blurred, and they accumulate into a blocky structure, and granular crystals are still the main form. The DSC of the quenched slag in molten water shows a crystallization exothermic peak, and the XRD diffraction pattern of the sample shows diffraction peaks of diopside and albite, indicating that the prepared glass-ceramics is still mainly in the glass phase, accompanied by the formation of crystals. The microscopic morphology changes of glass-ceramics are closely related to the doping amount of the smelting slag. As a nucleating agent, the presence of Zn^2+^ contributes to the formation of a small amount of calcium feldspar phase, thereby promoting the nucleation and crystallization of glass-ceramics [25]. Zn^2+^ effectively promotes the bulk crystallization of glass-ceramics by forming calcium feldspar nuclei [26]. With the increase in the doping amount of smelting slag makes the crystallization degree of glass-ceramics improves, and it tends to have uniform crystalline properties throughout the entire surface. The addition of smelting slag in excess will make the precipitation of feldspar increase, resulting in the consumption of a portion of Mg^2+^. Similarly, the precipitation of diopside consumes Mg^2+^. Based on the data from Figure 6, in samples S1 to S3, a decrease in SiO_2_ content from 6 wt.% to 0 wt.% leads to a reduction in particle size. In samples S5 to S7, an increase in the slag-to-ash mass ratio from 2 to 3 causes a decrease in particle size. Therefore, the increase in the doping amount of the smelting slag will inhibit the precipitation of diopside has been inhibited to a certain extent.

**Figure 7:**
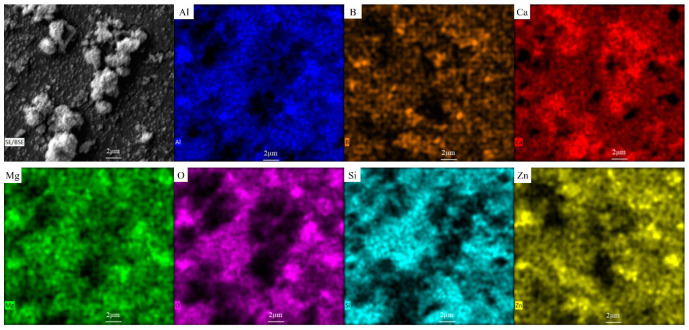
EDS images of glass-ceramics doped with 60% smelting slag.

The XRD pattern and SEM image analysis indicated that the complex and diverse composition structure of glass-ceramics mainly includes three types: diopside, calcium feldspar, and glass phase. To further investigate the impact of zinc-containing smelting slag on the elemental distribution during the preparation of slag glass-ceramics, EDS energy spectrum analysis was carried out. The face-scan elemental distribution map of glass-ceramics samples doped with 60% smelting slag is shown in Figure 7. In the figure, it can be seen that the glass phase significantly enriches Si, O, and Al elements; other elements are not prominently enriched. The reason for this phenomenon is that [SiO_4_] tetrahedra or [AlO_4_] tetrahedra serve as the structural units of the glass network core. Additionally, the Al content in the diopside phase is low, and the Ca and Mg contents are high. Therefore, combining the results of XRD experiments, it can be inferred that the blocky regions rich in Mg and Ca elements in the figure are a direct manifestation of the diopside phase. Observations show that there are clear enrichment zones of Ca and Al around the center of the diopside, which coincides with the characteristics of the calcium feldspar phase, which contains a small amount of Al and is rich in Ca. This confirms that this region is the calcium feldspar phase. The heavy metal Zn is distributed in various parts and is more abundant in the diopside and calcium feldspar crystals.

### 3.4. Raman Spectroscopy Analysis

**Figure 8:**
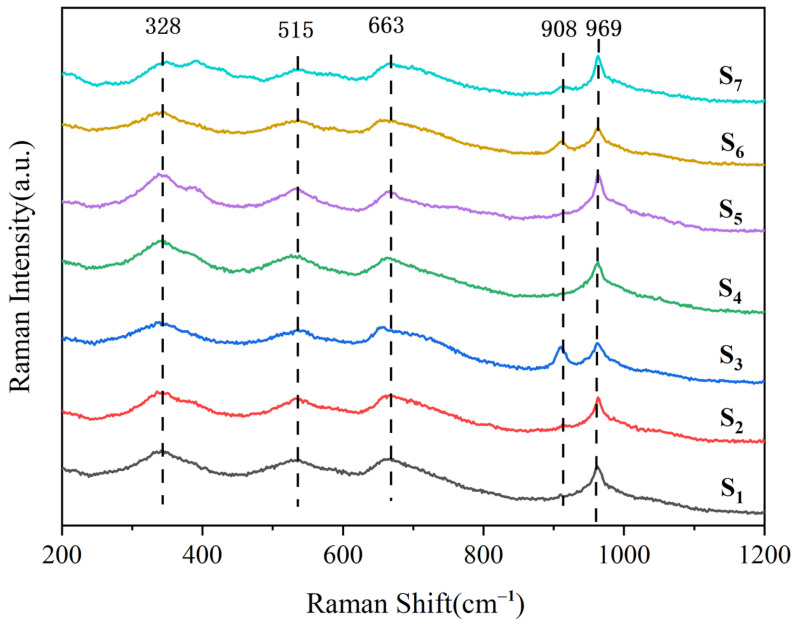
Raman spectra of the slag glass-ceramics with different content of smelting slag.

Raman spectroscopy is an effective characterization method to study the components and structural composition within glass [27]. As shown in Figure 8, the Raman spectra of all samples cover the wave number range of 200 to 1200 cm^−1^. Five main characteristic peaks can be observed from the Raman spectra. The Raman peak at 328 cm^−1^ is due to the bending vibration of the O–Si–O bond in the [SiO_4_] tetrahedron; the Raman shift at 515 cm^−1^ is anorthite phase, and this vibration is regarded as the symmetric stretching vibration resulting from the movement of oxygen atoms along the diagonal of the T–O–T bond angle; the Raman peak at 663 cm^−1^ is attributed to the displacement of the Si–O bridge oxygen bond in diopside the doping of iron. The Raman peaks at 908 cm^−1^ and 969 cm^−1^ are associated with the bending vibration of the Zn–O–Si bond [28] or the antisymmetric stretching vibration of the Si–O–Si bond. In the glass of doping a small amount of smelting slag, Raman spectra of slag glass-ceramics have a high consistency in peak position and shape. This phenomenon explains that Zn^2+^ and Mg^2+^ exist in the octahedron or tetrahedron in the same manner. The doping amount of smelting slag exceeds 40%, change occur in the absorption bands of the symmetric stretching vibration of the Si–O and Si–O–Si bonds, and Zn^2+^ enters the silicate network to form Zn–O–Si bonds [29]. Zn^2+^ as a network modifier can be well entered into the glass structure.

### 3.5. Physical Properties of Samples with Different Content of Smelting Slag

**Figure 9:**
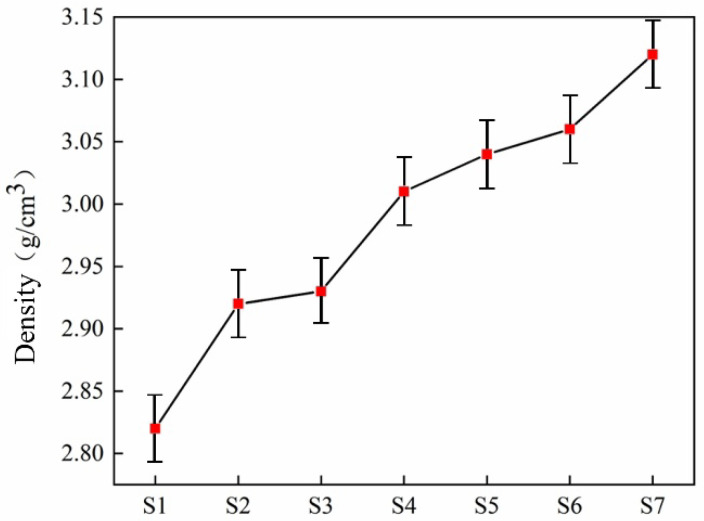
Density of the glass-ceramics with different content of smelting slag.

Table 4 shows the test results of physical properties such as density, Vickers hardness, and flexural strength of seven slag glass-ceramics samples with different zinc slag content. The microstructure of slag glass-ceramics determines the physical properties of materials. The basic composition of glass-ceramics is constant; the physical properties of glass-ceramics are mainly affected by the density, structure, and grain size of internal crystals. Density refers to the quality of substance contained in a unit volume, which largely depends on the chemical composition of the glass. Therefore, the study of glass density is helpful to characterize the compactness of the glass network, and then judge the structure change caused by composition change. The density test of glass-ceramics with different smelting slag doping amounts is shown in Figure 9. As the increase in doping amount of zinc-containing smelting slag increases, Zn^+^ content increases, cations of small radius can be filled in the interstices between the network, such as Zn^+^, although the connection break of silicon–oxygen tetrahedra does not cause the expansion of the network structure [30]. Consequently, the introduction of alkali metal oxides into silicate glass, such as ZnO, increases the structural compactness of slag glass-ceramics increases and the density also increases. The increase in smelting slag doping from 45.0% to 60.0%, the density increase in the sample is mainly due to the formation of high-density diopside phase and anorthite phase, which improves the microstructure.

**Figure 10:**
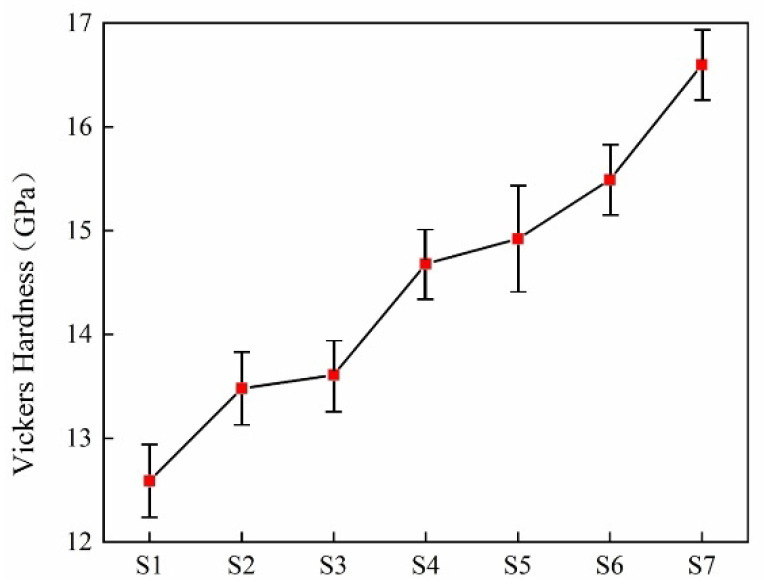
Vickers hardness of the glass-ceramics with different smelting slag doping amounts.

Vickers hardness refers to the ability of a material to resist local deformation, especially plastic deformation, indentation, and scratching. It is an indicator to measure the softness and hardness of a material. The harder the material, the smaller the indentation [31]. Microhardness is related to the crystal phase. The type and content of the crystal and the proportion of the residual glass phase are important factors influencing the hardness of glass-ceramics. The Vickers hardness of glass ceramics with different smelting slag doping amounts is shown in Figure 10. With the increase in smelting slag doping, diopside and anorthite phases are precipitated continuously, and the proportion of the glass phase decreases. Thereby, the Vickers hardness of glass-ceramics also showed an increasing trend.

**Figure 11:**
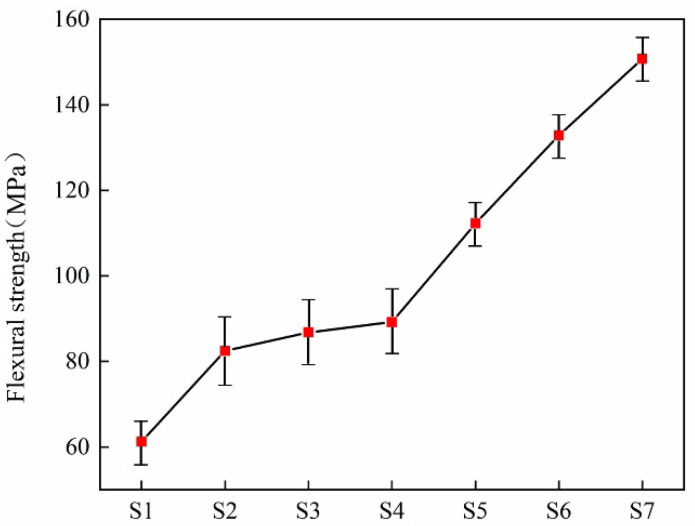
Flexural strength of glass-ceramics with different content of smelting slag.

Flexural strength is the ability of a material to resist fracture when subjected to a bending force. It is one of the important indices for weighing the mechanical properties of materials [32]. A high flexural strength means that the material is less likely to fracture when faced with a larger bending force. It depends on the crystal composition and microstructure of the glass-ceramics. As shown in Figure 11, the flexural strength of the slag glass-ceramics increases with the increase in the doping amount of zinc-containing smelting slag. Due to the continuous increase in Zn^+^ content, the improving crystallization degree of the glass-ceramics, the uniform distribution of fine and tight diopside crystals in the glass matrix, and the diopside phase belongs to the pyroxene group, present a chain-like structure within the silicate network. Accordingly, diopside with such a structure has favorable mechanical properties. As the increase in the doping amount of smelting slag promotes the precipitation of the anorthite phase, and the crystal morphology transforms from dense spherical crystals to interlocked blocky crystals, thereby improving the microstructure of the glass-ceramics. The generation of crystals with an appropriate amount increases the fracture energy of the samples, then enhancing their flexural strength.

### 3.6. Coefficient of Thermal Expansion

**Figure 12:**
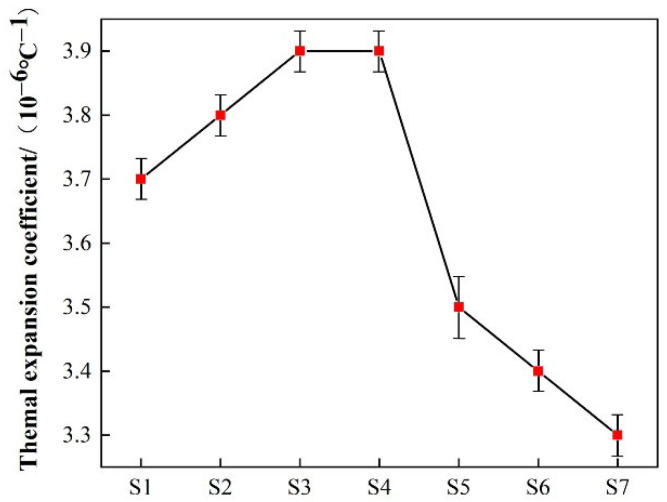
Coefficient of thermal expansion of samples S1~S7 at 600 °C.

Coefficient of thermal expansion refers to the coefficient that affects the size of an object due to temperature change. The ratio of the volume change in a substance because a change in temperature is called the coefficient of thermal expansion [33]. The characteristics of glass crystallization, especially the compactness of the microstructure, the type of crystalline phase, etc. All of them have a significant influence on the coefficient of thermal expansion of glass ceramics. The coefficient of thermal expansion results of the sample at 600 °C as shown in Figure 12. With the doping amount of smelting slag increasing from 30% to 60%, the coefficient of thermal expansion of the sample decreased from 3.7 × 10^−6^ °C^−1^ to 3.3 × 10^−6^ °C^−1^. The coefficient of thermal expansion increases with the increase in density when the doped amount of smelting slag is 30~45%. This is because the structure of the sample is tight and there are fewer voids inside, the distance between atoms is affected and becomes larger when the temperature increases, and there is no void to occupy, and the expansion becomes larger [34]. However, as the further increase in smelting slag doping amount, due to the coefficient of thermal expansion of anorthite phase is lower than the glass phase [35], the anorthite phase crystals increase gradually, and the coefficient of thermal expansion of samples changes from being determined by glass phase crystals to anorthite phase crystals, so the coefficient of thermal expansion of glass-ceramics shows a trend of decreasing gradually with the increase in smelting slag doping amount. In addition, while the increase in Al_2_O_3_ content, the content of [AlO_4_] tetrahedron in the system increases, which can enter the glass network structure and connect with [SiO_4_], and have repairing action for the fractured glass network, then improving the stability of the glass network structure, that causing the coefficient of thermal expansion of the glass-ceramics to decrease.

### 3.7. Study on Acid and Alkali Corrosion Resistance of Slag Glass Ceramics

Table 5 shows the acid and alkali corrosion resistance of the seven samples in this paper. The acid corrosion resistance of slag glass-ceramics increases gradually with the increase in slag doping amount, and the alkaline corrosion resistance changes little. Generally speaking, the corrosion resistance of slag glass-ceramics mainly depends on the ion exchange between metal ions in glass-ceramics and H- in acidic solutions [36]. The glass phase has a weak ability to cure and stabilize heavy metal ions; it is easier to be eroded by acid solutions than the crystalline phase, which shows poor acid resistance. In contrast, diopside and anorthite crystals exist stably in the crystal phase due to the formation of strong chemical bonds by metal ions, so they almost do not change significantly in the corrosion process [37]. This characteristic effectively prevents ion exchange between heavy metal ions and H- in acidic solutions. As the smelting slag doping amount increases, the proportion of the crystal phase increases, the proportion of the glass phase decreases, and the acid and alkali corrosion resistance of slag glass-ceramics increases, thus avoiding the possible environmental pollution problems.

### 3.8. Toxicity Leaching Test

Toxicity leaching tests are commonly used to characterize the effects of contaminants in materials containing hazardous substances on the environment. Table 6 shows the leaching concentrations of heavy metal zinc. According to the standard “Solid Waste Leaching Toxicity Leaching Method Sulfuric Acid-Nitric Acid Method” (HJ/T 299-2007) [20], the heavy metal concentrations in the leaching solutions of seven slag glass-ceramic samples in this paper were all below the standard limit (<100 mg/L). The slag glass-ceramic samples exhibited a good heavy metal curing ability. A comparison of samples with different smelting slag doping amounts found that the leaching concentration of heavy metals first increased and then decreased with increasing smelting slag doping amount. The curing heavy metal’s ability of slag glass-ceramic showed an increasing trend with the increase in smelting slag doping amount. The leaching rate of zinc is attributed to the heavy metal as a foreign impurity atom that enters the diopside and anorthite crystals and forms a substitutional solid solution [8]. The EDS experimental results indicated that the zinc element was relatively evenly distributed in the amorphous and crystalline phases, showing that a portion of the heavy metal zinc was stabilized and cured in the crystal phase, while another portion was cured in the amorphous phase. This found that the glass-ceramic curing method can effectively reduce the environmental impact of zinc. Therefore, with the increase in smelting slag doping amount, the proportion of the crystal phase increases, and the leaching rate of zinc decreases.

### 3.9. Heavy Metal Curing Mechanism of Slag Glass-Ceramics

**Figure 13:**
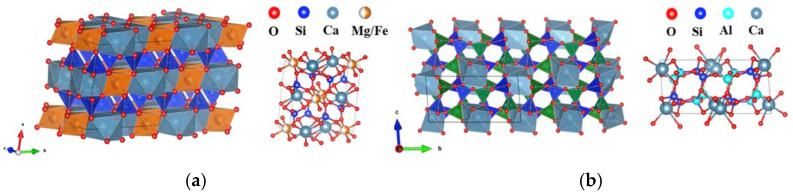
Cell structure of diopside and anorthite. (**a**) diopside; (**b**) anorthite.

In this paper, the heavy metal zinc is the main heavy metal in slag glass-ceramics. The XRD and Raman spectroscopy results show that diopside and anorthite are the primary crystal in the slag glass-ceramics, and the lattice constants tend to expand to two crystals, it may result in impurity defects [38]. The SEM-EDS analysis and toxicity leaching tests confirmed, that the presence of significant amounts of heavy metal Zn within the crystalline phase residues. These metal elements likely entered the crystal lattice, participating in the formation and growth of crystals, particularly diopside and anorthite crystal. Due to the glass-ceramics contain two crystal phases, the participation form of heavy metals within the crystal was analyzed through various factors, including ionic size, crystal structure type, and ionic valence state [39]. To understand the curing mechanism of heavy metal in the crystalline phases, firstly it is necessary to have a good understanding for the crystalline phase. Figure 13 is the crystal structures of anorthite and diopside [40]. As the figure show, the anorthite compose of silicon–oxygen tetrahedra [SiO_4_], aluminum–oxygen tetrahedra [AlO_4_], and irregular calcium–oxygen octahedra [CaO_6_] [38]. The aluminum–oxygen tetrahedra connect with silicon-oxygen where the Si/Al is approximately 1:1. The study indicated that the SiO_2_ content in anorthite is generally high, and the ratio than 1 [40]. The structure of diopside crystal is show in Figure 13. The cell body is composed of silicon–oxygen tetrahedra [SiO_4_], regular magnesium–oxygen/iron–oxygen octahedra [(Mg/Fe)O_6_], and irregular calcium–oxygen octahedra [CaO_6_] [41]. The silicon–oxygen tetrahedron is connected to the other two octahedra by the oxygen of the common vertex, and the magnesium–oxygen/iron–oxygen and calcium–oxygen octahedra are connected through edge of common vertices [41]. During the nucleation and crystallization stages of glass-ceramic formation, solid solutions often develop, with heavy metals enters the crystal as the foreign impurity atoms [41]. The most solid solutions formed within inorganic materials are substitutional, because solute atoms entered the crystal and replaced the normal lattice sites of original crystal. The formation of solid solutions by metal oxides in silicate materials primarily is the substitution between cations [40]. The size of the ions limits solid solution formation; from the perspective of crystal stability, the difference in ionic radius of the mutually substituted is less than 30% to form a substitutional solid solution [39]. Combined with the above factors and the cation metal radius obtained from the relevant literature, as shown in Table 7, the potential locations of heavy metals within the crystal were analyzed and are represented by impurity defect equations:

(1) $$ZnO\overset{CaAl_{2}Si_{2\;}O_{8}}{\to }Zn_{Al}^{\prime }+V_{\dot{Al}}+O_{o}$$

(2) $$ZnO\overset{Ca\left(Fe/Mg\right)\left(Si_{2}O_{6}\right)}{\to }Zn_{Fe}/Zn_{Mg}+O_{o}$$

**Figure 14:**
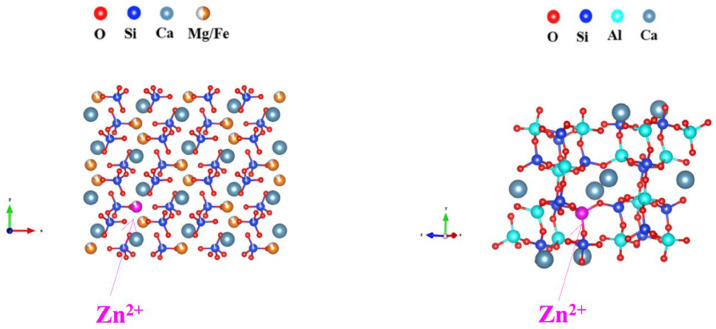
Structure of zinc in glass-ceramics.

Due to ionic radius differences, Zn^2+^ can only replace Al^3+^ in anorthite into the lattice. Because Zn^2+^ has a +2 valence, while Al^3+^ has a +3 valence, so the substitution of +3 valence of the Al^3+^ will generate positive charge vacancies at the original sites. The impurity reactions of heavy metal Zn in anorthite and diopside phases, as well as its location within the crystal, are show in Formulas (1) and (2) and Figure 14 [41]. Similarly, Zn^2+^ substitutes for Fe^2+^ or Mg^2+^ due to similar ionic radii, and both ions carry a +2 valance, so no charge vacancies are formed.

## 4. Conclusions

This paper adopts the traditional melting method to form base glass and uses zinc-containing smelting slag and fly ash as primary raw materials. Through a two-step method of nucleation and crystallization heat treatment, and CaO-MgO-Al_2_O_3_-SiO_2_ (CMAS) system glass-ceramics were successfully produced. The influence of different smelting slag doping amounts and the heavy metal zinc on the microstructure and properties of the glass-ceramics was studied. The following conclusions were obtained:

(1) Zinc as a network modifier can effectively enter the glass structure. As the doping amount of zinc-containing smelting slag continuously increases, the glass transition temperature and crystallization temperature decrease, which benefits the crystallization of the glass.
(2) The main crystalline phases in the glass-ceramics are diopside and anorthite. With the increase in the doping amount of smelting slag, the glass ceramics form a denser microstructure; at the same time, the density and acid and alkali resistance of the sample increase. The introduction of zinc significantly enhances the mechanical properties of the glass-ceramics. The maximum Vickers hardness reached 19.60 GPa, and the maximum flexural strength achieved was 160.75 MPa. The glass-ceramics exhibit excellent physical and chemical properties.
(3) The heavy metal zinc, whose content is high in the smelting slag, is cured in the amorphous and crystalline phases of the slag glass-ceramics as a solid solution, where Zn^2+^ substitutes for Al^3+^ and Fe/Mg^2+^ in the crystal. The slag glass-ceramics effectively cure heavy metal zinc, and the leaching concentration of zinc meets the requirements of the toxicity leaching standard, and the leaching rate tends to stabilize. This approach reduces environmental pollution and health risks while lowering disposal costs. The zinc-containing smelting slag is transformed into high-performance, non-toxic, and harmless glass-ceramic materials, achieving the resource utilization of solid waste.

## Figures and Tables

**Table 1:** Chemical composition of zinc-containing smelting slag.

Chemical Composition	SiO_2_	Al_2_O_3_	Na_2_O	MgO	K_2_O	CaO	TiO_2_	BaO	Cu	Cr	Zn	TFe
wt.%	26.65	14.29	5.32	3.97	0.40	29.78	0.25	0.51	1.04	0.71	2.36	14.72

**Table 2:** Experimental formula for zinc-containing smelting slag glass-ceramics (wt.%).

Specimen	Mass Fraction/%						
	Fly Ash	Smelting Slag	Quartz Sand	CaO	Na_2_CO_3_	Borax	CaF_2_
S1	45	30.0	6.0	8.0	2.5	8.2	5
S2	40	35.0	2.0	7.7	2.2	8.2	5
S3	35	40.0	0	7.5	2.1	8.2	5
S4	30	45.0	0	7.2	2.0	8.2	5
S5	25	50.0	0	6.9	1.8	8.2	5
S6	20	55.0	0	6.6	1.6	8.2	5
S7	20	60.0	0	6.3	1.4	8.2	5

**Table 3:** Characteristic temperatures and stability parameters of base glasses with different doping amounts of smelting slag.

Sample	T_g_/°C	T_c_/°C	ΔT/°C
S1	731	868	137
S2	726	863	142
S3	720	862	142
S4	710	847	137
S5	708	865	157
S6	698	867	169
S7	686	841	155

**Table 4:** Physical properties of glass-ceramics with different smelting slag doping amounts.

Sample	Density (g·cm^−3^)	Vickers Hardness (GPa)	Flexural Strength (MPa)
S1	2.82 ± 0.025	12.59 ± 0.4	61.25 ± 5.00
S2	2.92 ± 0.025	13.48 ± 0.4	82.45 ± 7.50
S3	2.93 ± 0.025	13.61 ± 0.4	86.8 ± 7.50
S4	3.01 ± 0.025	14.68 ± 0.4	89.2 ± 7.50
S5	3.04 ± 0.025	14.92 ± 0.6	112.25 ± 5.00
S6	3.06 ± 0.025	15.49 ± 0.4	132.86 ± 5.00
S7	3.12 ± 0.025	16.60 ± 0.4	150.75 ± 5.00

**Table 5:** Acid and alkali corrosion resistance of slag glass-ceramics with different smelting slag content.

Sample	Acid-Resisting (20% H_2_SO_4_)/%	Alkaline-Resisting (20% NaOH)/%
S1	73.24 ± 0.37	96.29 ± 0.48
S2	74.56 ± 0.37	97.35 ± 0.49
S3	76.91 ± 0.38	97.41 ± 0.49
S4	77.42 ± 0.39	97.66 ± 0.49
S5	79.36 ± 0.40	97.72 ± 0.49
S6	79.83 ± 0.40	97.76 ± 0.49
S7	80.96 ± 0.40	97.84 ± 0.49

**Table 6:** Leaching concentration of heavy metals in glass-ceramics.

Samples	S1	S2	S3	S4	S5	S6	S7
**Zn concentration** **(mg·L^−1^)**	4.42 ± 0.02	3.45 ± 0.17	3.82 ± 0.2	16.70 ± 0.08	32.00 ± 0.16	27.91 ± 0.14	24.15 ± 0.12

**Table 7:** Ionic radius of metals.

Metals	Mg	Al	Ca	Fe	Fe	Zn
Ionic radius (pm)	65	50	99	76	64	74

## Data Availability

The original contributions presented in this study are included in the article. Further inquiries can be directed to the corresponding author.
